# Femoral Vein Cannulation to the Left-Sided Inferior Vena Cava in Minimally Invasive Cardiac Surgery: A Case Report

**DOI:** 10.7759/cureus.82982

**Published:** 2025-04-25

**Authors:** Hiroto Kawakami, Takanobu Kimura, Hiroshi Tsuneyoshi

**Affiliations:** 1 Department of Cardiovascular Surgery, Shizuoka General Hospital, Shizuoka, JPN

**Keywords:** aortic valve replacement, cardiopulmonary bypass, congenital venous anomalies, femoral vein cannulation, left-sided inferior vena cava, minimally invasive cardiac surgery

## Abstract

A left-sided inferior vena cava (left-sided IVC) is a congenital venous anomaly in which the IVC runs along the left side of the abdominal aorta. We successfully inserted a venous cannula from the right femoral vein (FV) to the right atrium through a left-sided IVC. We established cardiopulmonary bypass during minimally invasive cardiac surgery (MICS).

We present a case of a 75-year-old man with controlled diabetes mellitus who underwent aortic valve replacement via MICS (MICS-AVR) with a 23-mm Epic porcine valve. He had severe aortic regurgitation with a three-month history of exertional dyspnea. MICS-AVR was planned as he wished to return to physical labor early. However, a left-sided IVC was found on the preoperative computed tomography. It exhibited a peculiar flexure, raising concerns about whether the venous cannula would pass through it. However, FV cannulation was successfully performed under X-ray fluoroscopy.

This case demonstrates the feasibility of FV cannulation even in patients with left-sided IVC, with X-ray fluoroscopy proving useful in avoiding venous injuries and enhancing the reproducibility of the procedure. It also highlights the importance of preoperative assessment for IVC anomalies to prevent perioperative complications and optimize the surgical plan.

## Introduction

A left-sided inferior vena cava (left-sided IVC), a congenital venous anomaly in which the IVC runs along the left side of the abdominal aorta, occurs in approximately 0.2%-0.5% of the total population [1,2]. The formation of the IVC during embryonic development involves the regression and fusion of several veins. The left-sided IVC arises when the left supracardinal vein persists and the right supracardinal vein regresses. The left-sided IVC normally passes in front of or behind the descending aorta at the level of the left renal vein and then ascends the right side before entering the right atrium (RA) [1,2]. The left-sided IVC seldom ascends on the left side of the abdominal aorta, traverses the diaphragm, converges with the azygos vein, and enters the RA [2,3].

Cardiopulmonary bypass in minimally invasive cardiac surgery (MICS) often involves femoral vein (FV) cannulation through the IVC. However, FV cannulation in patients with a left-sided IVC has not been reported. We performed aortic valve replacement (AVR) by MICS (MICS-AVR) in a patient with aortic regurgitation (El Khoury Functional Classification type I) [4], and a left-sided IVC was identified during the preoperative assessment. FV cannulation was performed from the right FV through the left-sided IVC using a 23-Fr single-stage cannula.

## Case presentation

**Figure 1:**
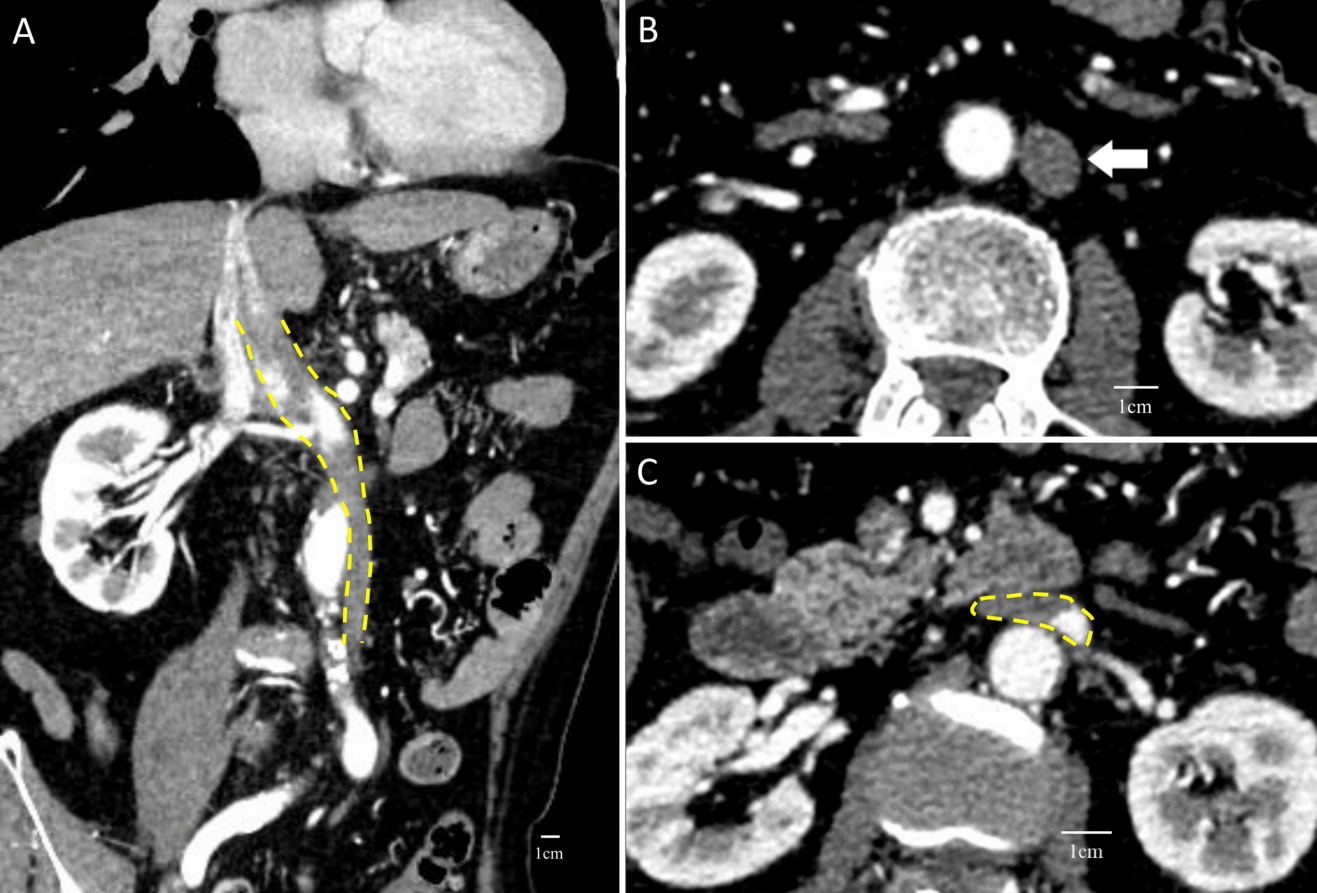
Left-sided IVC on enhanced CT. (A) Cross section including the IVC (yellow dashed line). (B) IVC (white arrow) on the left side of the aorta. (C) Crossing of the aorta and IVC (yellow dashed line) IVC: inferior vena cava; CT: computed tomography

A 75-year-old man with controlled diabetes mellitus presented with a three-month history of exertional dyspnea. His B-type natriuretic peptide level was elevated, and echocardiography revealed severe aortic regurgitation with left ventricular enlargement. Consequently, the patient was referred to us for cardiac surgery. MICS-AVR was planned as he wished to return to physical labor early. However, a left-sided IVC was identified on the preoperative enhanced cardiac computed tomography (CT). The IVC ascended along the left side of the retroperitoneum before joining the left renal vein. It then crossed in front of the descending aorta and merged with the right renal vein, running along the right side and ultimately entering the RA (Figures 1A-1C).

**Figure 2:**
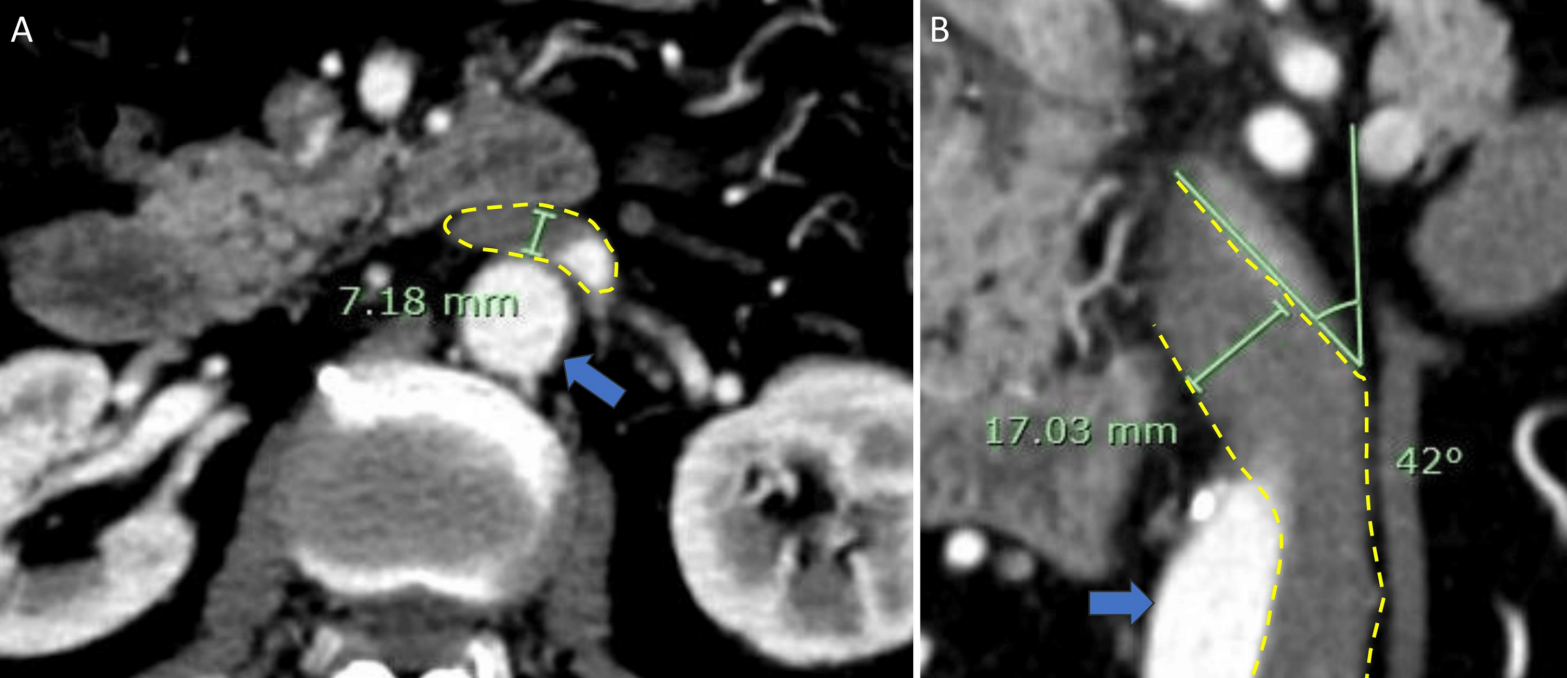
The diameter and degree of bending at the flexure. (A) The diameter of the flexure part of the left-sided IVC (yellow dashed line) on the axial image. The blue arrow indicates the descending aorta. (B) The diameter and degree of bending at the flexure part of the left-sided IVC (yellow dashed line) on the coronal image. The blue arrow indicates the descending aorta IVC: inferior vena cava

MICS-AVR is typically performed with a single FV cannulation [5], but concerns arose about whether a venous cannula could pass through the flexure of the IVC in this patient. Direct placement of a venous cannula into the RA through thoracotomy was considered, but this would have required a larger chest incision, which was preferably avoided. The flexure measured approximately 7 mm × 17 mm (Figure 2A), with a bending angle of about 40° on the CT image (Figure 2B).

A 23-Fr single-stage cannula was planned for FV cannulation. If the FV cannula failed to pass, the plan included adding right internal jugular vein (IJV) cannulation. The right FV was chosen because no compression was observed in the bilateral common iliac veins, and right FV cannulation was a familiar technique for the surgical team.

**Figure 3:**
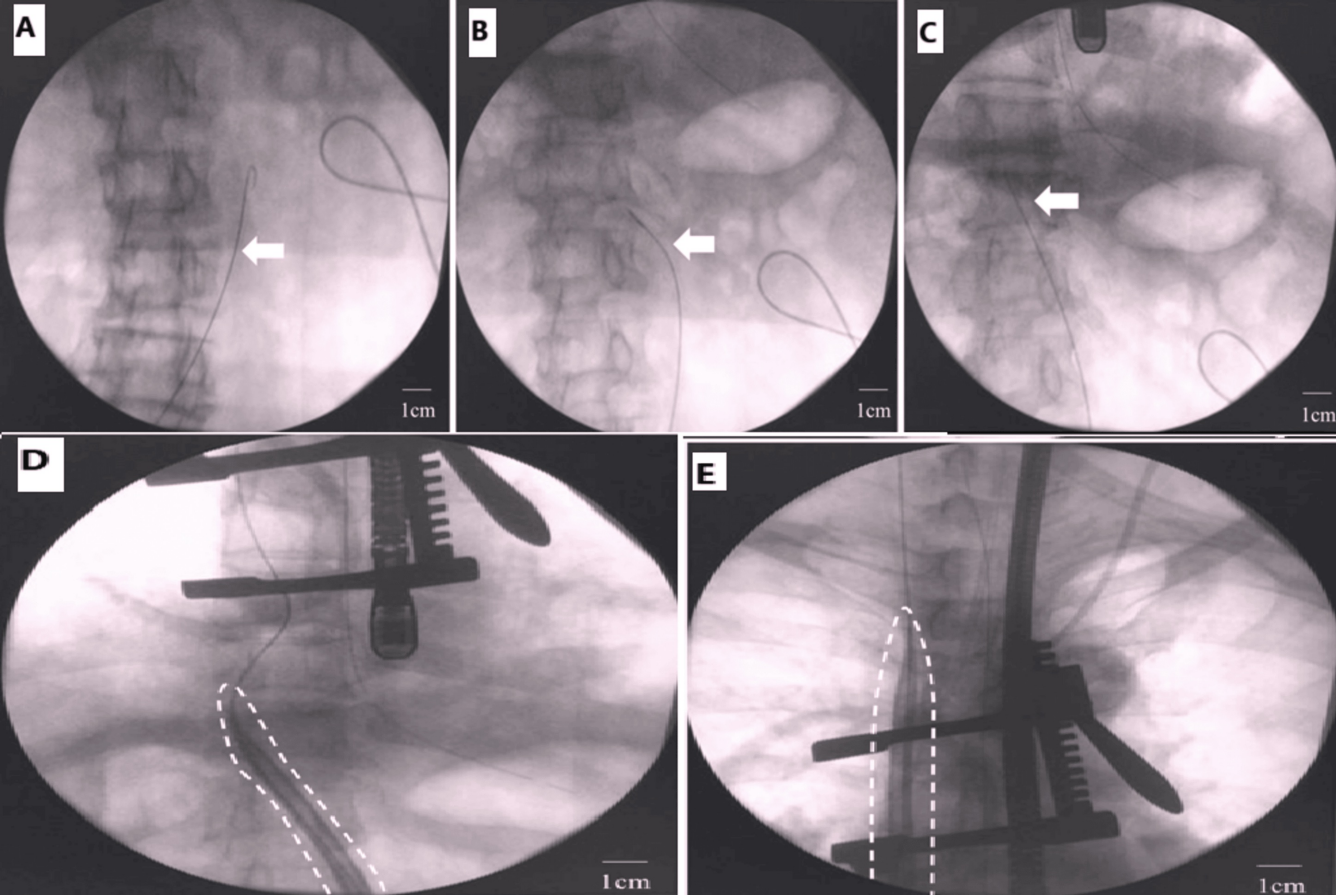
FV cannulation under X-ray fluoroscopy. (A,B) Guide wire (white arrow) advancing into the left-sided IVC and (C) unexpectedly straightening through the flexure. (D) Venous cannula (white dashed line) passing through the flexure and (E) reaching the junction of the SVC and the RA FV: femoral vein; IVC: inferior vena cava; SVC: superior vena cava; RA: right atrium

During the operation, a sheath was placed in the right IJV preoperatively as a precaution. With the patient in the supine position, a 6-cm thoracotomy was made in the third right intercostal space. An arterial cannula was inserted into the femoral artery after exposing the right femoral vessels and performing systemic heparinization. A guidewire was then carefully advanced from the right FV into the IVC under X-ray fluoroscopy to prevent venous injury. The wire passed smoothly through the IVC flexure, straightening more than expected (Figures 3A-3C). It reached the superior vena cava (SVC) via the RA. A 23-Fr single-stage cannula (HLS cannula, Getinge, Sweden) was inserted along the guidewire, smoothly ascending through the IVC to the SVC (Figures 3D, 3E).

A cardiopulmonary bypass was initiated, achieving full flow with a single right femoral venous drainage and eliminating the need for IJV cannulation. After establishing cardiopulmonary bypass, an 18-Fr left ventricular vent was inserted through the right superior pulmonary vein, and an aortic root cannula was placed. Cardiac arrest was induced with antegrade extracellular-type cardioplegia. The aortic valve was replaced with a 23-mm Epic porcine valve. Weaning from cardiopulmonary bypass was uneventful, and the venous cannula was removed smoothly. The aortic clamp time was 93 minutes, the cardiopulmonary bypass time was 149 minutes, and the total operation time was 266 minutes. No complications occurred. Postoperative recovery was uneventful, and the patient was discharged on postoperative day 8.

## Discussion

This case demonstrates two crucial clinical findings. Even in the presence of a left-sided IVC, FV cannulation was successfully performed under fluoroscopic guidance. Additionally, when using FV cannulation in cardiopulmonary bypass, it is important to preoperatively confirm any anatomical variations in the IVC.

First, under X-ray fluoroscopy, FV cannulation was successfully performed through the left-sided IVC despite initial concerns about the left-sided IVC flexure. Physicians should consider FV cannulation even in patients with left-sided IVC, especially in MICS, which often prefers FV cannulation for establishing cardiopulmonary bypass [5]. To the best of our knowledge, there are no reported cases of FV cannulation through a left-sided IVC in cardiopulmonary bypass, and only one case [6] of cardiopulmonary bypass in a patient with a large IVC (LIVC) has been reported. Schoephoerster et al. [6] reported a case of thoracoabdominal aortic aneurysm repair in a patient with left-sided IVC, where FV cannulation was avoided due to concerns about IVC injury. On the other hand, in some cases [7,8], guidewires and catheters have passed through the flexure of the left-sided IVC. Olagunju et al. [7] reported a case of transcatheter atrial septal defect closure in a patient with LIVC, where a guidewire could not advance from the right FV. The subsequent venogram revealed a left-sided IVC. Then, a Rosen wire (Cook Medical, Bloomington, IN) and a Cobra catheter (Cook Medical) were advanced into the RA through the left-sided IVC under X-ray fluoroscopy. Sardi and Minken [8] reported a case of placing an intracaval filter in a left-sided IVC. The filter was placed in the IVC below the left renal vein through the right IJV, and it successfully passed the flexure of the left-sided IVC via the guidewire under fluoroscopic guidance. From these cases, it is evident that guidewires, catheters, and other equipment can pass through the flexure of the left-sided IVC. Our case particularly demonstrates that a venous cannula can also pass through it. However, without fluoroscopic guidance, this may not be successful [8]. X-ray fluoroscopy should be utilized to enhance the reproducibility of FV cannulation in left-sided IVC and to avoid venous injury. Second, preoperative assessment for IVC anomalies is essential when planning FV cannulation [9,10]. In our case, LIVC was identified on preoperative CT images, and the venous cannula was carefully advanced from the FV under X-ray fluoroscopy to minimize the risk of IVC injury. If necessary, a venous sheath was preoperatively placed in the right IJV to prepare for double venous drainage. In some cases, FV cannulation is attempted without screening for IVC anomalies [9,10]. Singh et al. [9] reported a case of MICS using FV and IJV cannulation without preoperative identification of an interrupted IVC, a congenital anomaly in which the IVC is discontinuous in the middle and does not merge into the RA [1,2]. After a vein presumed to be the IVC was snared, the abdomen became tense and distended due to the unintentional snaring of the hepatic vein confluence. In another case of MICS in a patient with an interrupted IVC [10], the guidewire from the right FV stopped midway. The absence of X-ray fluoroscopy left the cause unclear, ultimately forcing the surgeons to switch to median sternotomy to establish cardiopulmonary bypass. Preoperative identification of IVC anomalies is crucial when performing FV cannulation to prevent serious complications and unplanned changes in the operative plan. Our case especially emphasizes the importance of preoperatively identifying left-sided IVC. Additionally, left-sided IVC is often misdiagnosed as left para-aortic lymphadenopathy on CT images [11-13], so careful attention is necessary.

## Conclusions

In our recent case of MICS-AVR in a patient with a left-sided IVC, a venous cannula was successfully inserted via the right FV. This demonstrates the feasibility of FV cannulation even in the presence of a left-sided IVC. Although FV cannulation through a left-sided IVC has not been previously reported, physicians should consider FV cannulation in patients with LIVC to establish cardiopulmonary bypass. X-ray fluoroscopy is valuable for improving the reproducibility of the cannulation procedure and preventing venous injury. Furthermore, preoperative assessment of IVC anomalies is crucial to minimize risks and optimize the surgical plan.
